# Digital cities and the spread of COVID-19: Characterizing the impact of non-pharmaceutical interventions in five cities in Spain

**DOI:** 10.3389/fpubh.2023.1122230

**Published:** 2023-03-23

**Authors:** Jorge P. Rodríguez, Alberto Aleta, Yamir Moreno

**Affiliations:** ^1^Instituto Mediterráneo de Estudios Avanzados (IMEDEA), CSIC-UIB, Esporles, Spain; ^2^Institute for Biocomputation and Physics of Complex Systems, University of Zaragoza, Zaragoza, Spain; ^3^Department of Theoretical Physics, University of Zaragoza, Zaragoza, Spain; ^4^CENTAI Institute, Turin, Italy

**Keywords:** epidemic spreading, digital twins, COVID-19, non-pharmaceutical interventions, pandemic control

## Abstract

Mathematical modeling has been fundamental to achieving near real-time accurate forecasts of the spread of COVID-19. Similarly, the design of non-pharmaceutical interventions has played a key role in the application of policies to contain the spread. However, there is less work done regarding quantitative approaches to characterize the impact of each intervention, which can greatly vary depending on the culture, region, and specific circumstances of the population under consideration. In this work, we develop a high-resolution, data-driven agent-based model of the spread of COVID-19 among the population in five Spanish cities. These populations synthesize multiple data sources that summarize the main interaction environments leading to potential contacts. We simulate the spreading of COVID-19 in these cities and study the effect of several non-pharmaceutical interventions. We illustrate the potential of our approach through a case study and derive the impact of the most relevant interventions through scenarios where they are suppressed. Our framework constitutes a first tool to simulate different intervention scenarios for decision-making.

## 1. Introduction

The COVID-19 pandemic has globally impacted a plethora of systems, with health (1), socio-economic (2, 3), and environmental (4) consequences. To control the spread of SARS-CoV-2, policymakers implemented a diversity of procedures, grouped into either mitigation or suppression strategies. Lockdowns, implying home confinement, were frequently introduced to stop the spreading in early 2020 when the dynamics of the infection mechanisms were not clear. However, these lockdowns resulted in deep impacts on the economy, and later on, other non-pharmaceutical interventions were designed, such as the use of face masks, the closure of restaurants, universities, or schools, as well as contact tracing, testing, and isolation of close contacts of infected individuals.

The initial stages of the pandemic represented a high degree of uncertainty, both regarding the original transmission of the pathogen to human beings and reliable surveillance data [due to low testing efforts and inappropriate surveillance systems (5)]. Nowadays the situation has improved, as the availability of more data—even if many times of poor quality and low reliability—in principle allows to characterize the spreading at a large scale. Moreover, the existent data enables the development of mathematical models that help quantify the observed evolution of the pandemic and evaluate the effects of the intervention scenarios.

The first wave of COVID-19 raised a challenge for modeling approaches due to the general bad data quality. Specifically, the lack of knowledge about the COVID-19 spread, the similarity between the symptoms of COVID-19 and those of influenza, and the low testing effort led together to lower rates of diagnosis and hence underreporting mainly in the number of cases (6), but also in the number of deaths. Seroprevalence studies (7) and the analysis of anomalies on the temporal series of deaths (8) were needed to estimate the real impact of the spreading process, showing that there were up to 10 times more cases than the reported ones. In this regard, spreading models can shed light on the real outcome of the infection across the population.

To properly model the spreading of a disease in the population, it is fundamental to acknowledge that human interactions are highly heterogeneous. Although network epidemiology can capture part of this diversity, such as the broad nature of the distribution of the number of interactions, the variability of contexts remains out of this formalism. These contexts can be effectively captured using multilayer networks, which are networks with multiple layers, each one describing the interactions in a different context (9, 10). In this work, we leverage anonymous, publicly available data to build high-resolution synthetic cities and encode them in multilayer networks (11, 12). We use these synthetic networks to study the propagation of the first wave of COVID-19 in five Spanish cities. Furthermore, we extend the simulation to the second wave for the particular case of the city of Zaragoza and thoroughly characterize the impact of non-pharmaceutical interventions during this period.

## 2. Materials and methods

### 2.1. Multilayer contact networks

**Figure 1:**
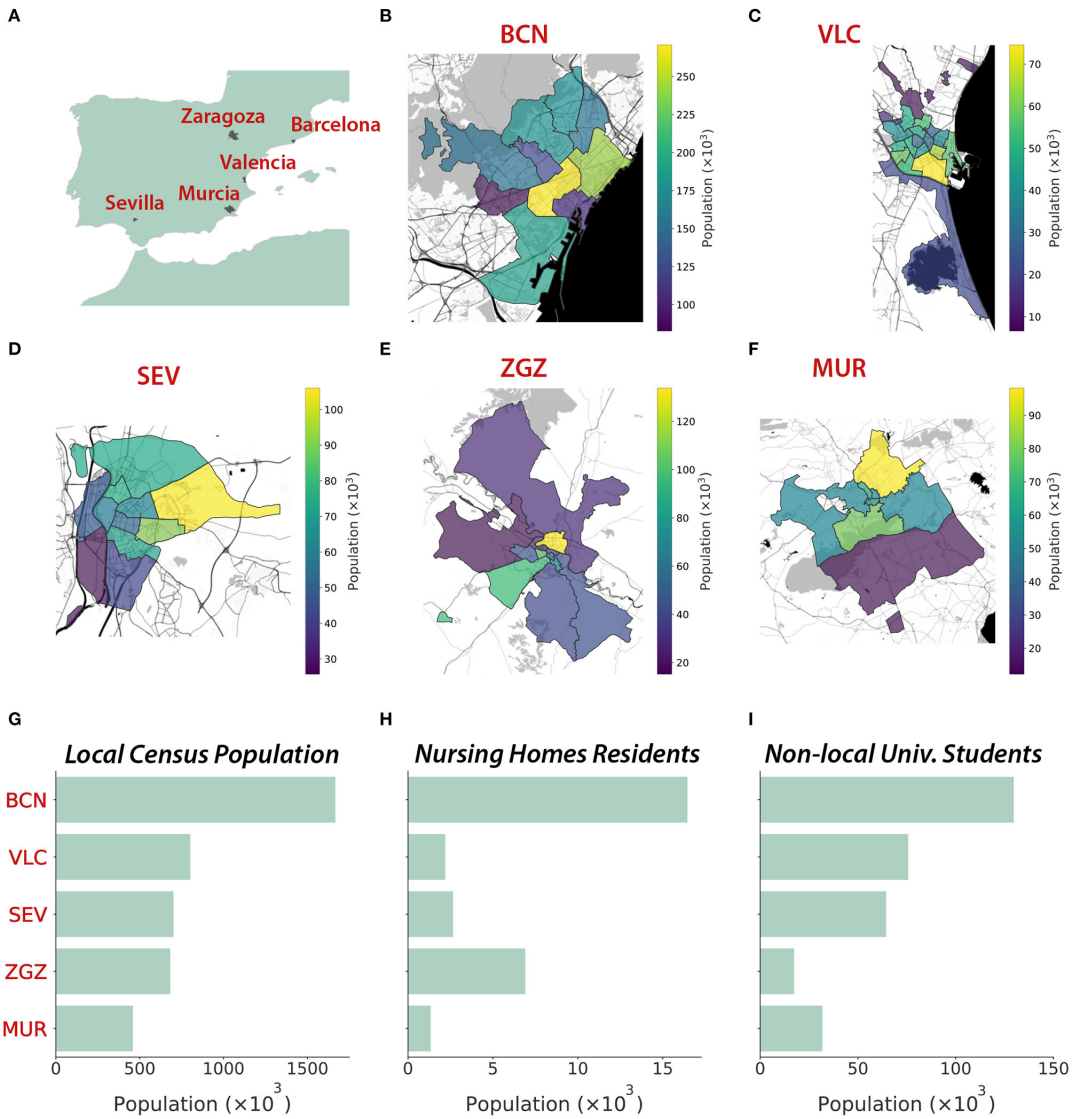
Geography and demography of the five cities represented through synthetic populations. **(A)** Location of the five cities. **(B–F)** Geographical representation (Map tiles by Stamen Design, under CC BY 3.0. Data by OpenStreetMap, under ODbL) of the district structure at each city (source: Instituto Nacional de Estadística, INE), with colors representing the population of each district. **(G)** Local and non-local populations (nursing homes residents and university students). The five cities studied are Barcelona (BCN), Valencia (VLC), Seville (SEV), Zaragoza (ZGZ), and Murcia (MUR).

We create five digital populations describing the inhabitants and the interactions between them in the cities of Barcelona, Valencia, Seville, Zaragoza, and Murcia, all of them located in Spain (Figures 1A–F). Their population ranges between 450 thousand and 1.7 million inhabitants (Figures 1G–I). Additionally, we include external individuals that may not be registered in the census but with most of their interactions expected to happen in these cities. These external individuals include old people living in nursing homes and non-local university students. Each inhabitant is represented in the population as a node connected to other inhabitants. These links were built according to the specific data sources for each city and each feature, as listed in the Supplementary material.

#### 2.1.1. Demography

We obtained the geographical distribution, sex, and age of the inhabitants of the cities at the beginning of 2020 from multiple demographic data sources. The maximum spatial resolution was the census district (Figures 1B–F), at which we found most of the needed information to create the synthetic digital cities. Ages were available in age groups with a resolution of 5 years. Thus, we interpolated these age groups to consider a resolution of 1 year between 0 and 30 years, which was necessary to properly infer the interactions at schools and universities. This allowed us to create a synthetic population for each city resembling the characteristics of the real ones.

#### 2.1.2. Contact networks

**Figure 2:**
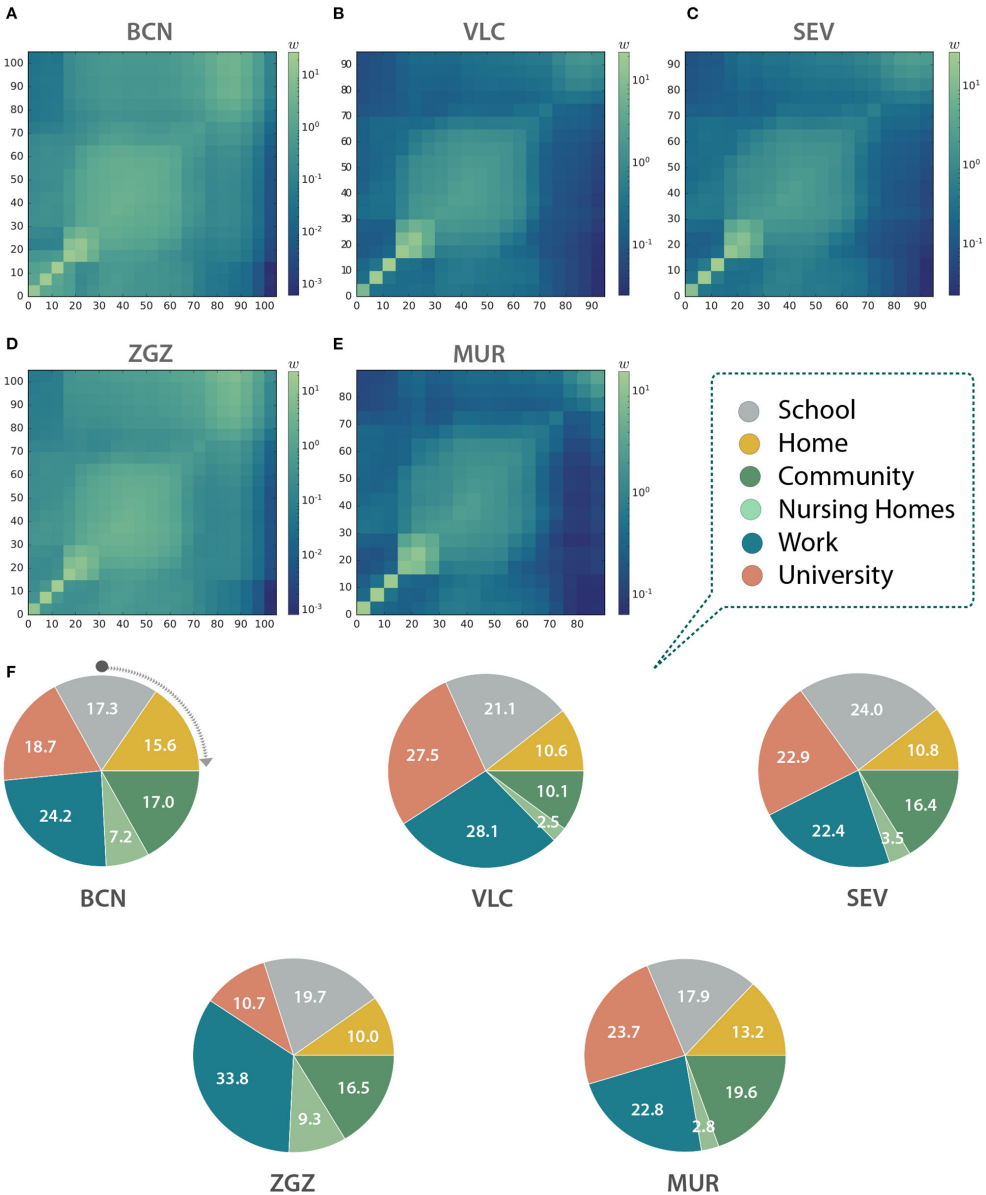
Contact matrices between age groups in **(A)** Barcelona (BCN), **(B)** Valencia (VLC), **(C)** Seville (SEV), **(D)** Zaragoza (ZGZ), and **(E)** Murcia (MUR). **(A–E)** Each entry *w*_*ij*_ is computed as the total number of observed links between individuals belonging to the age group in row *i* and column *j*, normalized with the number of individuals in each column. Thus, they represent, for a given column, the expected number of links of a random individual to individuals from each row. **(F)** Link distribution among the different layers in the five studied cities expressed as a percentage of the total number of contacts in each city.

We modeled the contacts between individuals through networks described by the aggregation of multiple interaction layers (13). Specifically, we considered six interaction layers: home, nursing homes, school, work, university, and community. We incorporated empirical data from multiple datasets available from national, regional, and local sources to infer the connections between the individuals in the different environments introduced by the interaction layers. In Figure 2, we show the age mixing patterns of the population extracted from our synthetic cities (12).

#### 2.1.3. Home layer

Individuals in the home layer are connected if they live together. We extracted the information on the number of homes of a specific size, the average home size, and the home structure at the district level. We use the information from the national census of 2011 (14), the most recent one that is currently available, (see also the Supplementary material) for all the cities except for Barcelona, for which this information is available from local sources with higher resolution. We also use the age difference of the home nuclei, at district resolution, from the national census. This information is key for reproducing realistic home contact matrices, as the home structures include in the “adults” category any individual aged between 25 and 64 years old. Connecting randomly pairs of individuals in this broad group could lead to less representative links in most homes, composed of two adults alone or with children or old people. As we do not know if these nuclei are assortative or disassortative, we include the data on the age difference of the nuclei to create this synthetic layer.

#### 2.1.4. School layer

This layer connects all the students and a teacher within the same scholar unit. Besides, all the teachers that work in the same center are also connected. We included in this layer the infant levels (0–3 and 3–6 years old), primary school (7–12), secondary school (13–16), high school (17–18), and job training (from 17). We inferred these connections using data on the number of students per level, the number of units per level, and the number of schools, taking into account the levels offered by each kind of school. This information was available at the district level for Barcelona and Valencia. Additionally, data on the specific size of each specific unit at each center was available for Valencia and used for that layer inference. For Seville, Zaragoza, and Murcia, this information was available at the municipality level, so we mapped the school coordinates to the districts, and we inferred the rest of the needed information from the one at the municipality level. Once the synthetic units, at each center, were created, we assigned individuals from the population to those units. First, we filled each unit with individuals of that specific age that have their homes located in the same district. Secondly, the units that had not been filled totally with individuals from the same district were filled with individuals from other districts, with a priority determined by the distance between the centroids of the districts, until all the units in the city were full. We assumed that, after that step, the remaining individuals were not included in the education system. Teachers were chosen randomly among individuals aged between 30 and 70 from any district in the city.

#### 2.1.5. University layer

We generated the university contact layer using the national statistics of the number of registered students per university and per degree, split by sexes, available from the Spanish Ministry of Education, considering both undergraduate and graduate programs, for the academic year 2019–2020. We considered the universities located in the same province as the studied cities, after removing distance-learning universities. Then, we estimated which students are registered and live in the city, and which ones are external, either registered in the same or another province. Local students have also interactions in the other layers, while externals are only in the university layer. Finally, we obtained the national age profiles by sex of university students, picked individuals from our synthetic population, and introduced those external, according to these profiles. We designed a connectivity pattern of all-to-all for degrees that had sizes lower than 50 people, and otherwise generated patches with all-to-all connectivity of a maximum size of 50 people for the larger ones.

#### 2.1.6. Work layer

In the work layer, individuals that belong to the same company are connected together. We obtained the distribution of companies' sizes (S) throughout all the Spanish provinces, which follows a power-law distribution pdf (*S*)~ *S*^−2^. Then, we generated companies with sizes that follow this distribution, and, when sizes were higher than 20 people, we distributed the workers among patches with a maximum size of 20 people. We estimated the number of workers by subtracting the number of autonomous workers from the number of registered workers in each city, according to the Social Security reports. We extracted sex and age features also from Social Security reports on a national scale. We did not consider as potential workers those that were assigned a school patch, either as teachers or students. The synthetic companies were filled with individuals from the synthetic population following the corresponding distributions by age and gender.

#### 2.1.7. Nursing homes layer

We collected information on the number of nursing homes and their capacity in each municipality. Additionally, we gathered national statistics on the age and gender of the people that reside in nursing homes. We assumed that the nursing homes need one caretaker for every four places, and chose that uniformly from those in the dataset older than 16 years old (minimum age for being allowed to work). Inside each nursing home, we assumed an all-to-all connection. Note that individuals residing in nursing homes do not interact in the household layer.

#### 2.1.8. Community layer

We generated a synthetic community layer connecting randomly pairs of individuals living in the same district, according to the contact matrices for Spain in Prem et al. (15). There were contact matrices available for home, work, school, and other locations, and we chose the latter. This dataset reported the probability of connecting pairs of individuals according to their ages, in age groups of 5 years up to 75 years old. For individuals older than 75 years old, we extrapolated the data of the oldest available group.

### 2.2. Epidemic spreading

#### 2.2.1. Spreading model

We used the COVASIM software for modeling the spread of COVID-19 (16). COVASIM is an open-source Python-based agent-based modeling tool. COVASIM considers a susceptible-exposed-infected-recovered or dead (SEIRD) epidemic model that includes disease parameters informed by the medical literature. The infected compartment is divided into asymptomatic and symptomatic infectious individuals, with the latter including presymptomatic, mild, severe, and critical stages. The three symptomatic stages can evolve to the recovered state, while the critical state can alternatively lead to the death of the individual (Supplementary Figure S1). The probabilities of developing symptoms, severe symptoms, a critical case, and from it the death of the individual are specified by age groups, arranged in 10-year-long age cohorts. This software has been used for studying different scenarios, for example, assessing the test-trace-quarantine strategy (17) or quantifying the risk of outbreaks after international border opening (18). We modified COVASIM to include the specific details of our synthetic cities. Specifically, we included the age, sex, and contacts of the individuals in each of the considered cities. We ran independent simulations where each simulation chose one randomly infected seed as the first infected individual. Then, we kept the endemic realizations, defined as those leading to a finite number of deaths, which we set higher than 10 for the first wave. For the second wave, we also requested that there were more than 10 death observations in the last 10 days of the realization. Apart from the internal parameters of COVASIM, we considered independently the infection rate and the date of arrival of the first infected individual. We calibrated both parameters for each city analyzing the official time series of deaths (see section 2.2.2), which were more reliable than the number of cases that could suffer from high underdetection rates (6).

#### 2.2.2. Epidemic data

We obtained the temporal series of the number of deaths and the number of confirmed cases from the Spanish Ministry of Health (19) at the province level, with daily resolution. Then, we multiplied these values by the fraction of the province's population living in the city. We averaged the rescaled data over a moving window of 7 days (the specific day ± 3 days) to smooth the fluctuations.

### 2.3. Quantifying public health policies

To illustrate the potential of our approach, after calibrating the first wave of COVID-19 in our model with synthetic digital cities, we implemented a case study of the second wave focusing on the city of Zaragoza. This second wave occurred between July and December 2020.

The non-pharmaceutical interventions that were introduced in this city to mitigate the spread of COVID-19 were the following:

- Testing and tracing. Positive tested individuals and their close contacts were isolated for 14 days until 30th September, and from then on for 10 days.
- Restrictions on restaurants, cafes, and nightlife. Starting on 5th August, lifted on 4th September, and re-started on 19th October.
- Opening of the schools with reduced group sizes and safety measures. Different levels started progressively, from 7th September to 17th September.
- Opening of the universities with reduced group sizes. The university was opened on 14th September.
- Interventions impacting the community layer. We considered the interventions related to the State of Alarm and those related to the capacity and schedules of restaurants and bars.

In addition to these policies, we also took into account the annual leave period of workers and considered a reduced number of interactions in the work layer starting on 15th July until the middle of September, with the maximum reduction on 1st August. In terms of the model, this implies a higher amount of time (e.g., more weight of this layer) associated with the interactions in the community layer.

We used the infection rate (β) and date of arrival of the infected seed obtained in the calibration of the first wave and ran the model from the estimated arrival of the first case (see section 3.2) to 1st December 2020. Since we ran the simulations from the beginning of the first wave, we lifted progressively the restrictions that were active from the 14th of March in two steps: on the 1st and 20th of June, following the progressive lift of the restrictions that actually happened.

We kept 25% of the contacts in the school and university layers to simulate the small group's policy, and reduced β by 50% in the school layer, considering the strict protocol to avoid contagion at schools.

After calibrating the model of the second wave with these non-pharmaceutical interventions and considering the results of the simulation, we introduced different counterfactual scenarios in which we quantified the effect of each of the non-pharmaceutical interventions adopted. More specifically, we computed the number of deaths and the disease prevalence by performing simulations with the same epidemiological parameters but switching on and off alternative interventions. Finally, we computed the relative change in the relevant quantity *X* (*X* = deaths or prevalence) as *r* = (*X*^counterfactual^ − *X*^simulated-2nd-wave^)/*X*^simulated-2nd-wave^. Therefore, the absolute change can be obtained as *X*^counterfactual^ = (1 + *r*) · *X*^simulated-2nd-wave^.

We considered the following nine different counterfactuals:

- No testing and no contact tracing. The testing intervention was removed. Hence, as contact tracing depends on the results of the testing process, contact tracing was automatically removed.
- No contact tracing. To analyze the impact of the contact tracing strategy and decouple it from the testing process, we kept the testing intervention and its parameters but removed contact tracing interventions.
- Opening 100% university. We considered the opening of the university layer with 100% of the contacts, instead of the 25% contacts estimated through the small group's intervention.
- Not opening university. We simulated a scenario where the university layer remained closed.
- Opening all schools together (x2). The school opening was done following a staggered strategy, such that each level started on a different date. We simulated scenarios where all the levels started on the same date, either on the date of the earliest opening (7th September) or the latest opening (17th September).
- 100% β in schools, whole groups. Schools were one of the sectors where strong protocols were introduced, reducing considerably the infection rate and also the group size. We simulated the absence of these protocols, keeping the same infection rate as in the rest of the layers, and considering this layer with whole groups, that is, 100% of the contacts.
- Not opening schools. We quantified the changes in the outcome of the second wave if the schools had not been open.
- No interventions in October. We observed that the interventions in October were key to controlling the second wave. Thus, we removed these interventions and computed this counterfactual, keeping the same final date, such that the result was comparable with the rest of the counterfactuals. However, we assumed that removing these interventions would imply that the second wave continued growing on time. To characterize this growth (in terms of both time extent and outcome), we ran additional simulations for 15 and 30 days more and compared them with extrapolations of the original second wave simulation for the same period (without including additional measurements introduced on December 2020 or calibrating the observed data in that period).

## 3. Results

### 3.1. Contact matrices

With the information contained in the multilayer networks we can infer the contact matrices of the population (12). These matrices can be used to inform classical epidemiological models for studies not based on agent-based models, or to obtain an aggregated picture of the interactions in the system, as in this case. Indeed, as we can see in Figures 2A–E, the shape of the matrices indicates that our networks display an assortative pattern with blocks of infants, adults, and the older adults with a higher preference to interact with other individuals with similar ages. The number of contacts per layer is also significantly different both within and across cities (Figure 2F). For instance, workplace contacts are predominant in Barcelona and Zaragoza, the university ones in Valencia and Murcia, and the school contacts in Seville. Note that our agent-based model explicitly contains each link between two individuals, and thus these matrices are not used to model the spreading.

### 3.2. First wave

**Figure 3:**
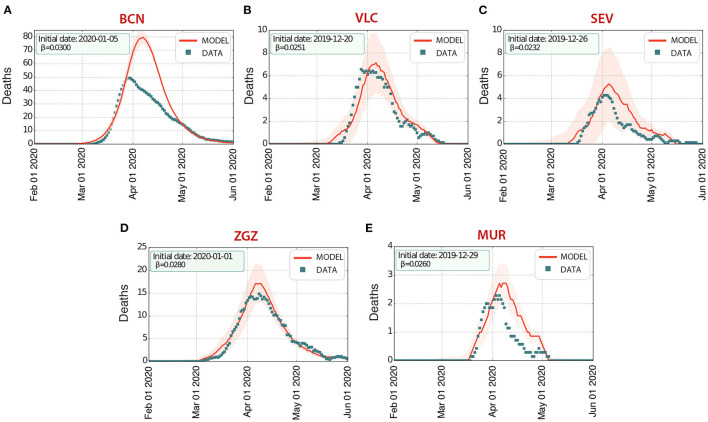
First wave of infections of COVID-19 in five Spanish cities: **(A)** Barcelona (BCN), **(B)** Valencia (VLC), **(C)** Seville (SEV), **(D)** Zaragoza (ZGZ), and **(E)** Murcia (MUR). The number of deaths *D* estimated by our model (solid line, the shaded area represents the 5-95% CI) agrees with the data (properly rescaled, see section 2) reported by the Spanish Ministry of Health (dots), with an excess of deaths in the initial stages of the wave.

In order to be able to explore realistic counterfactuals for the effectiveness of the most important NPIs adopted, we started by simulating the first wave to calibrate the model for each of the cities considered. Specifically, we ran simulations of the spread of COVID-19 in these cities using the software COVASIM. We estimated the transmission rate and the arrival of the initial seed, considered as a single infected individual (Figure 3, Table 1). Our multilayer approach allowed us to introduce the effects of the national lockdown declared on 14th March 2020, reducing the contacts in the work layer to 20% (10% for Barcelona) and 0% in the university, school, and community layers. Our results indicate an earlier arrival of COVID-19 to these cities (upon the assumption of a single initial seed), and they highlight the earlier occurrence of deaths at the beginning of the first wave, not considered in the official statistics, in Barcelona, Valencia, Seville, and Zaragoza. The prevalence estimates from our model are compatible with those obtained from the nationwide seroprevalence study in Spain (see Table 1). Note that this seroprevalence study detected 10 times more cases than the ones reported by the surveillance system.

**Table 1:** Starting date (Start), infection rate (β), prevalence (Prev.), and number of deaths (Deaths) from the model output of the first wave.

**City**	**Start**	**β**	**Prev. (5–95% CI)**	**Deaths (5–95% CI)**	**Ref. Prev. (5–95% CI)**
Barcelona	Jan 05	0.0300	14.6 (13.1–16.1)	2,334 (2,188–2,477)	7.4 (6.2–8.9)
Valencia	Dec 20	0.0251	3.1 (1.5–4.7)	227 (141–313)	2.1 (1.5–3.0)
Seville	Dec 26	0.0232	2.4 (1.1–4.7)	195 (75–314)	2.7 (1.9–3.8)
Zaragoza	Jan 1	0.0280	6.1 (3.9–8.3)	599 (465–733)	5.2 (3.9–6.9)
Murcia	Dec 29	0.0260	2.4 (1.6–3.3)	89 (67–111)	1.6 (1.0–2.5)

The reference prevalence (Ref. Prev.) is that provided in the first phase of the national study of seroprevalence for the first wave in its second round, finished on June 1st (7).

### 3.3. Second wave. Counterfactuals

**Figure 4:**
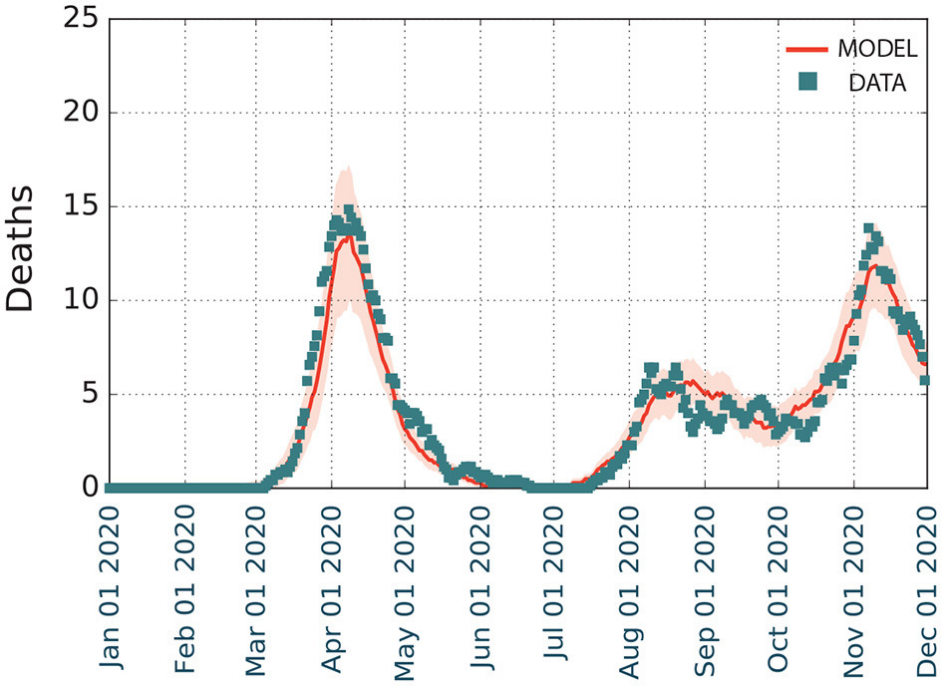
Modeling the first and second waves of COVID-19 spreading on Zaragoza from January to December 2020. We represent the temporal evolution of the number of deaths *D*, with shaded regions depicting the 5–95% confidence intervals of the model.

When restrictions were progressively lifted after the end of the first wave, a second wave started growing (Figure 4), and we calibrated our model to obtain the impact of the interventions on our model parameters (see section 2).

The calibration of the second wave led to the following results:

- Varying number of links in the community layer: connections were set at 50% of the baseline value (1st June, progressive lift of restrictions), 80% (20th June, end of the national State of Alarm), 50% (5th August, regional limits on restaurant and bar schedules), 100% (4th September, lift of restrictions), 30% (19th October, regional limits on the schedule and capacity of restaurants and bars), and 10% (26th October, national State of Alarm).
- Varying the weight of the links in the community layer: increase by 50% from 20th June to 26th October (end and beginning, respectively, of the national State of Alarm).
- Varying the number of links in the work layer: connections were set at 70% of the baseline value (1st June), 50% (15th July), 30% (1st August), and 50% (20th September). These changes in summer accounted for the summer holidays period. Mobility reports (20) showed a slow return to mobility associated with work in September.
- Testing. The probability of symptomatic individuals being tested (per day with symptoms) was estimated to be 15% between 1st July and 14th September, and 9% from 15th September. The delay between the test and the result notification (with the beginning of the associated isolation period) was fixed to 1 day.
- Contact tracing. The contacts from positive-tested individuals were traced with a general probability *P*_*t*_. Additionally, *P*_*t*_ was weighted for each layer of contacts, fixing the weights 1 for home, 0.8 for school, 0.6 for university, 0.8 for work, 0.0 for the community [until the introduction of the contact tracing app Radar COVID (21), which increased it to 0.05], and 0.0 for nursing homes. We fixed the time between the positive notification and the communication with close contacts to 2 days. *P*_*t*_ was estimated to be 0.4 between 1st July and 19th August, 0.45 between 20th August (introduction of Radar COVID) and 30th September, and 0.5 from 1st October (extra support to contact tracing from trained soldiers).

Overall, the model (Figure 4) estimated that there were 1,354 deaths (5–95 CI), with a prevalence of 22.6% (21.0–24.2% 5–95 CI). This prevalence was higher than the reported in the fourth phase of the national seroprevalence study carried out in mid November (7), which estimated a prevalence of 12.7% (10.1–15.8% 5–95 CI) at the province level. Even though the data at the municipality level is not available, it reported that the prevalence in municipalities with more than 100,000 inhabitants was 50% larger than in the smaller ones. The province of Zaragoza is highly heterogeneous in terms of size, with one municipality (out of 293) containing 69% of the almost 1,000,000 inhabitants in the province. Thus, it is expected that the prevalence at the city level should be much larger. Similarly, our model in the first wave agreed with the empirical observations of the temporal evolution of the number of deaths documented, with a minor overestimation for Murcia but a larger one for Barcelona. We interpret these divergences as possibly missing data, in line with other studies that have claimed a higher number of deaths than that reported by the official statistics, which was particularly significant in the administrative region of Catalonia, where Barcelona is located (8).

**Figure 5:**
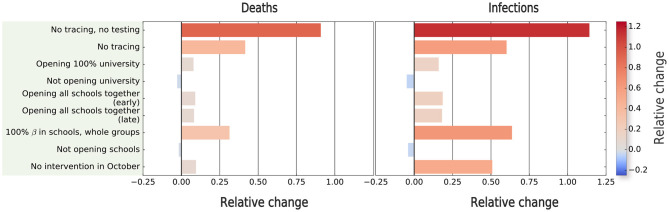
Estimating the impact of non-pharmaceutical interventions on the second wave of COVID-19 spreading in Zaragoza. We compute the relative change with respect to the outcome of the model in terms of prevalence and number of deaths for different simulated scenarios. The relative difference in the relevant quantity *X* (*X* = deaths or prevalence) is computed as (*X*^counterfactual^ − *X*^simulated-2nd-wave^)/*X*^simulated-2nd-wave^.

The results of the counterfactual analysis shown in Figure 5 indicate that the combination of tracing and testing, with the associated isolation of positive individuals, was very effective in reducing the number of both deaths and infections. Note that for this case, the counterfactual (i.e., lack of such measures) led to more than twice the number of infected individuals, and also nearly doubled the number of deaths. Next, we quantified the impact of contact tracing alone by keeping the testing process, together with the isolation of individuals with a positive test, but removing the contact tracing. This scenario also showed an increase in both the number of infections and deaths. However, the increase of both observables was twice lower than if both contact-tracing and testing are removed, highlighting the importance of combining these two interventions to achieve the best result. The third-most important counterfactual according to the increase in deaths was the removal of the interventions in schools, which however produce the second-largest increase in the number of infections, but with a smaller impact in the number of deaths because most infections would occur in non-risk age groups. Interestingly, opening the university without restrictions would lead to fewer infections than with schools completely opened, however leading to more deaths. The synchronous opening of the schools for different levels also implied an increase in prevalence and deaths, but with a lower impact than other interventions. Finally, there were also some counterfactual scenarios that produced a decrease in the observables, such as keeping schools or the university closed. However, their impact was minor.

For the sake of completeness, we also assessed the impact of the interventions in October to control the outbreak. In principle, these measures did not have a big impact as shown in Figure 5. However, the interpretation is not straightforward, because our second wave simulations finish on December 1st, and the absence of these interventions may have implied a later end of this wave. To quantify this, we extended, without adding any new intervention, both the calibrated and counterfactual simulations, finishing the simulation on (a) 15th December and (b) 31st December. Our results showed that the relative change between the real extrapolated framework and this counterfactual kept increasing after 1st December (9.5% for deaths, 50.9% for prevalence), as the extrapolated values were higher on 15th December (40.3% for deaths, 71.1% for prevalence), and slightly decreased at 31st December (25.3% for deaths, 66.5% for prevalence), indicating that by this date, the counterfactual wave would have finished. Hence, this extrapolation suggests that this counterfactual would have implied, compared with the rest of the counterfactuals, the second-largest increase in prevalence, and the fourth-largest increase in the number of deaths.

## 4. Discussion and conclusions

The quick spread of a deadly infection among a population represents a threat to public health systems, which requires immediate action and extra resources in order to mitigate and eventually control the impact of the associated disease on the population. However, interventions need to be carefully evaluated, as our society is a complex interdependent system in which mitigating the effects of one disease might result in non-desirable side effects such as the reduction of the services provided to both prevent and treat other diseases (22–24). To avoid these effects, scientists and policy-makers need computational resources that allow for a fast assessment of the possible outcome of interventions whenever pharmaceutical interventions, such as vaccination campaigns, are not possible. This is often the case when new diseases emerge, as seen with the virus SARS-CoV-2, which currently represents one of the major threats to public health systems. On the one hand, governments and health organizations need to allocate significant resources in order to develop and test vaccines and/or specific pharmacological treatments. On the other hand, traditional surveillance methods are likely to miss large numbers of new infections in the population, leading to a high number of undocumented infections during the early stages of the disease, as also happened in the case of SARS-CoV-2 (25). In such situations, non-pharmaceutical interventions represent the alternative to at least earning time. This is where data-driven and computational frameworks are fundamental to inform models that can illustrate the outcome of different non-pharmaceutical interventions. In this paper, we have presented a model that could be used to characterize the consequences of a plethora of non-pharmaceutical interventions. We applied the model to study the first and second waves of COVID-19 in Spain, finding that testing, tracing, and isolation were among the most effective interventions to reduce both the number of deaths and infections, in line with similar studies for other geographical locations (13, 26, 27). Our study also shows that, on the whole, the interventions adopted during the second wave for the city of Zaragoza, were effective and reduced the number of deaths and infections by around 10% and 50%, respectively. The effort presented in this work, informing a computational model of COVID-19 spreading with synthetic populations based on real data, has the potential to speed up the analysis of different intervention scenarios in future large-scale epidemic emergencies.

This work has some limitations that deserve further discussion. First of all, our simulations considered a single randomly chosen initial seed, and from this, we estimated the date of arrival of the disease. Nevertheless, the spreading process could have started by the arrival of several infected individuals either synchronously or asynchronously. However, we think that these approaches are equivalent, as they would lead to the same number of infected individuals at later dates. In contrast, different effects could emerge when specific individuals, according to, for example, their age, district of residence, or employment status, display a higher likelihood to introduce the infection. Another limitation is the isolation of the cities, as they are considered closed systems. This can be solved by introducing a spontaneous infection rate reflecting the imported cases from other locations, although we assume that in the cases of generalized local transmission this rate would lead to minor differences. The emergence of variants with different infection and recovery rates and death probabilities is challenging for these models, requesting the parameter correction for subsequent waves happening when other variants were present. The latter, however, does not impact predictions at the early stages of an emerging disease, which is when evaluating possible NPIs is most needed. Finally, we considered the main non-pharmaceutical interventions that were applied in the city of Zaragoza, but their calibration may include the effect of other interventions that we assumed to have minor effects.

Our multilayer network method, informed from multiple data sources, contrasts with other approaches used for modeling the spread of COVID-19. These include the introduction of meta-population approaches representing recurrent mobility (28, 29), the use of demography to infer social contact data (6), information from real-time human mobility indices (30), or the use of high-resolution individual trajectories (13). We acknowledge that the latter method would be the ideal scenario in terms of accuracy, but it would request the availability of detailed mobility data, which is not directly linked to layers whose dynamics are shaped by interventions. When such data is not available, our method can inform mathematical models of spreading while keeping realistic social contact data.

In summary, our work shows how models of digital cities can be coupled to agent-based epidemiological models of disease dynamics and be used for scenario evaluation. Our approach aligns with the spirit of developing digital twins to face the challenges raised by the Sustainable Development Goals (https://sdgs.un.org), related for example with environmental problems or health issues. After the extensive data search needed for creating these cities (see Supplementary material), updating these digital cities will be a more straightforward task, allowing them to timely inform the models that help design non-pharmaceutical interventions to mitigate the effects of future pandemics.

## Data availability statement

The data sources are specified in the Supplementary material. The code used for simulating the first and second waves in Zaragoza is available in the following URL: https://github.com/jorgeprodriguezg/digicovid. Digital Cities are available from JR (jorgeprodriguezg@gmail.com) upon reasonable request.

## Author contributions

JR, AA, and YM contributed to conception and design of the study and analyzed the results. JR collected, cleaned, organized the data, performed the statistical analysis, and wrote the first draft of the manuscript. All authors contributed to the article and approved the submitted version.
