# Supplementary material for: Digital cities and the spread of COVID-19: Characterizing the impact of non-pharmaceutical interventions in five cities in Spain
Source: Front Public Health. 2023 Mar 23;11:1122230. doi: 10.3389/fpubh.2023.1122230 (PMC10076648; doi:10.3389/fpubh.2023.1122230)
Supplement: Supplementary file 1 [file Data_Sheet_1.PDF]

Digital cities and the spread of COVID-19: characterizing the impact of non-pharmaceutical interventions in five cities in Spain

Jorge P. Rodríguez, Alberto Aleta, Yamir Moreno

SUPPLEMENTARY MATERIAL

SUPPLEMENTARY FIGURES

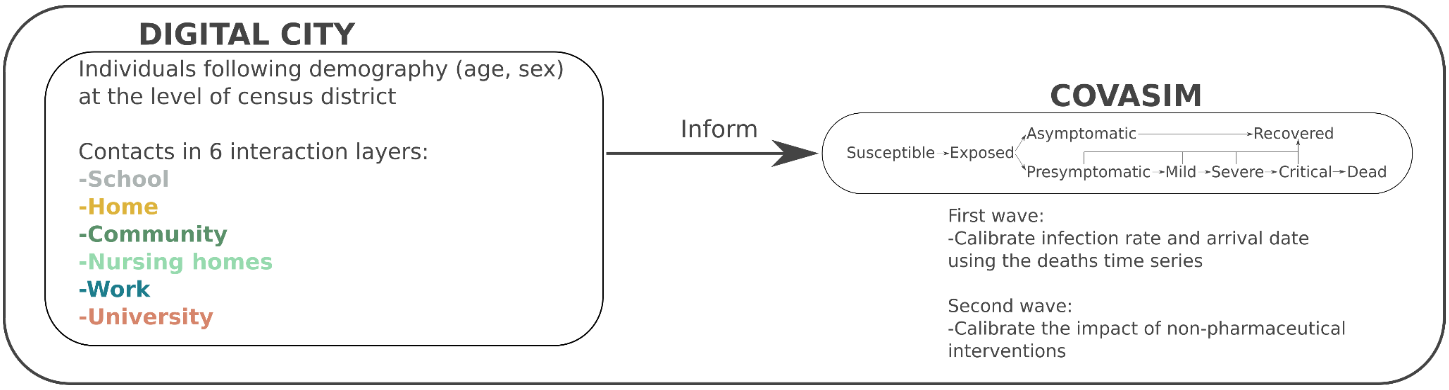

Fig. S1. Workflow including the main components of this study: the digital cities inferred from multiple data sources and the modeling software COVASIM.

DIGITAL CITIES: DATA SOURCES

Data available from scientific literature

-Contact matrices for 152 countries (data obtained: Spain, other locations, used for inferring community layer):
https://journals.plos.org/ploscompbiol/article?id=10.1371/journal.pcbi.1005697#sec020

Data available from national sources

-Instituto Nacional de Estadística, population by age and sex, home sizes and home structure, at district level:
https://www.ine.es/censos2011/tablas/Inicio.do
-Registered students at university, per degree and university:
https://www.ciencia.gob.es/portal/site/MICINN/menuitem.7eeac5cd345b4f34f09dfd1001432ea0/? vgnextoid=0930dd449de8b610VgnVCM1000001d04140aRCRD
-Statistics of registered students at university, BsC (data obtained: ages):
http://estadisticas.mecd.gob.es/EducaDynPx/educabase/index.htm?type=pcaxis&path=/Universitaria/Alumnado/Nueva_Estructura/GradoCiclo/Matriculados/&file=pcaxis -Statistics of registered students at university, MsC (data obtained: ages):
http://estadisticas.mecd.gob.es/EducaDynPx/educabase/index.htm?type=pcaxis&path=/Universitaria/Alumnado/Nueva_Estructura/Master/Matriculados/&file=pcaxis -Age of registered students at job training programs (used data from course 2018-2019):
http://estadisticas.mecd.gob.es/EducaDynPx/educabase/index.htm?type=pcaxis&path=/Educacion/ Alumnado/Matriculado/2018-2019RD/RGCiclosFP&file=pcaxis&l=s0
-Private request to the Ministry of Universities (Spain) on the number of registered students at university per municipality (BsC and MsC).
-Instituto Nacional de Estadística, number of companies per region and number of employees: https://www.ine.es/jaxiT3/Tabla.htm?t=298&L=0
-Instituto Nacional de Estadística, national employment survey, information at municipality level (using data at the first term of 2019):
https://www.ine.es/dyngs/INEbase/es/operacion.htm?c=Estadistica_C&cid=1254736176918&menu=resultados&idp=1254735976595 -Number of affiliated employees to the National Social Security, by municipality (using data at the last day of January 2019):
https://www.seg-social.es/wps/portal/wss/internet/EstadisticasPresupuestosEstudios/Estadisticas/EST8/EST167/21b04ced-f0f1-4bb9-9662-0437a6f9934e/29f824da-afbf-4af2-aa3e-9c8751e86991 -Instituto Nacional de Estadística, age and gender of residents in nursing homes:
https://www.ine.es/jaxi/Tabla.htm?path=/t20/e244/colectivos/p02/l0/&file=02005.px&L=0
-Envejecimiento en red (CSIC), number of places and nursing homes per municipality:
http://envejecimiento.csic.es/documentos/recursos/residencias/

Data available from regional/local sources

1. Barcelona

-Population by age and sex, at district level:
https://www.bcn.cat/estadistica/castella/dades/tpob/pad/padro/a2020/edat/edatq03.htm
https://www.bcn.cat/estadistica/castella/dades/tpob/pad/padro/a2020/edat/edatq04.htm
-Number of school students, number of units and number of centres at district level, data

obtained for course 2019-2020, for different levels:
https://educacio.gencat.cat/ca/departament/estadistiques/estadistiques-ensenyament/cursos anteriors/curs-2019-2020/

2. Valencia
-Population by age and sex, at district level:
http://www.valencia.es/ayuntamiento/catalogo.nsf/IndiceAnuario?readForm&lang=1&capitulo=2&tema=2&bdOrigen=ayuntamiento/estadistica.nsf&idApoyo=58FB3C7A3D56E414C1257DD40057EB6C
-Students registered in each unit (per level) in school programs, by centre: https://dadesobertes.gva.es/es/dataset/edu-alu-gen-2020
-Students registered in each unit in job training programs, by centre:
https://dadesobertes.gva.es/es/dataset/edu-alu-fp-2020
-List of scholar centres, including their geographical coordinates (updated 21 September 2020):
http://www.ceice.gva.es/es/web/centros-docentes/descarga-base-de-datos

3. Seville
-Population by age and sex, at district resolution:
https://www.sevilla.org/servicios/servicio-de-estadistica/datos-estadisticos/explotacion-estadistica padron#autotoc-item-autotoc-0
-Number of students, number of units and number of centres per level per municipality (3. Sociedad, 3.1 Enseñanza y formación):
https://www.juntadeandalucia.es/institutodeestadisticaycartografia/badea/informe/anual? CodOper=b3_151&idNode=23204
-List of scholar centres, including their geographical coordinates:
https://www.juntadeandalucia.es/datosabiertos/portal/dataset/directorio-de-centros-docentes-de andalucia#csv

4. Zaragoza
-Number of students, number of units and number of centres, per level per municipality: https://www.aragon.es/-/ensenanzas-de-regimen-general
-List of scholar centres, including their geographical coordinates:
https://opendata.aragon.es/datos/catalogo/dataset/directorio-de-centros-educativos-de-aragon

5. Murcia
-Population by age and sex, at section resolution (districts are composed by sections):
https://econet.carm.es/web/crem/inicio/-/crem/sicrem/PU_padron/p20/sec23_sec24_31.html
https://econet.carm.es/web/crem/inicio/-/crem/sicrem/PU_padron/p20/sec24_sec25_31.html -Number of students, number of units and number of centres, per level per municipality:
https://econet.carm.es/web/crem/inicio/-/crem/sicrem/PU_EDUCNOUNIV/Indice2016.html -List of scholar centres, including their geographical coordinates:
https://murciaencifras-com.carto.com/tables/centros_educativos_1/public
